# Prevalence of Chlamydia trachomatis in Women Who Are Candidates for In Vitro Fertilization in a Private Reference Service in Southern Brazil: A Cross-Sectional Study

**DOI:** 10.7759/cureus.24109

**Published:** 2022-04-13

**Authors:** Rafaela A Link, Carlos A Link, Matheus H Benin Lima, Bruna W Pasetti, Ricardo F Savaris

**Affiliations:** 1 Obstetrics and Gynecology Department, Universidade de Passo Fundo, Faculdade de Medicina, Passo Fundo, BRA; 2 Surgical Sciences, Universidade Federal do Rio Grande do Sul, Postgraduate Program in Medicine, Porto Alegre, BRA; 3 Reproductive Endocrinology and Infertility, Clínica PROSER, Porto Alegre, BRA; 4 Obstetrics and Gynecology Department, Universidade Federal do Rio Grande do Sul, Porto Alegre, BRA

**Keywords:** rt-pcr (real time - reverse transcription polymerase chain reaction), tubal infertility, prevalence study, chlamydia, in vitro fertilization (ivf)

## Abstract

Introduction

*Chlamydia trachomatis* (CT) has been related to fallopian tube damage and infertility. Its prevalence in the population that attend public services is known; however, there is scant data on this factor in private infertility clinics. The objective of this study is to verify the prevalence of CT among women attending a private in vitro fertilization (IVF) reference clinic in southern Brazil.

Methods

This is a cross-sectional study carried out between January 1, 2019, and August 30, 2021, at an IVF private clinic in southern Brazil. Infertile women between 18 and 50 years old, who provided a morning urinary sample for reverse transcription-polymerase chain reaction* *(RT-PCR) test for CT analysis, were included in the study. The variables studied included the patient's age, body mass index (BMI), duration of infertility, type of infertility, indication for IVF, and detection or not of CT in the urine.

Results

The prevalence of CT was 10.84% (22 out of 203; 95% CI: 7.27-15.87). Patients with secondary infertility were older and had more ovarian and tubal factors compared to cases of primary infertility. The tubal factor was the most prevalent (27.3% in women with primary infertility and 35.8% in those with secondary). Time of infertility and BMI were similar between groups. Our results are derived from a single private IVF clinic which reduces the external validity.

Conclusion

The prevalence of 10.84% of CT in this population raises the importance of screening for sexually transmitted infections for proper treatment and to achieve better IVF outcomes.

## Introduction

Infertility affects up to 12% of couples of reproductive age worldwide [1,2]. The tubal factor is one of the most common causes of female infertility [3]. Tubal damage has been related to *Chlamydia trachomatis* (CT), a sexually transmitted infection. The World Health Organization estimated the global prevalence of chlamydia was 3.8% (95% CI: 3.3-4.5) among women in 2016 [4]. This bacterium affects women of reproductive age with a prevalence of 4%, and 70% of these infections are asymptomatic [5]. These asymptomatic women are left untreated and are prone to CT-related sequelae, such as infertility, due to the possibility of the bacterium ascending through the upper female genital tract, infecting the fallopian tubes and causing salpingitis, leading to functional damage of the tubes and infertility by tubal factor [6,7]. 

There are different methods of identifying and diagnosing CT infection; however, the collection of urine samples and the use of the reverse transcription-polymerase chain reaction (RT-PCR) technique have been considered the test of choice for chlamydia and have replaced culture as the diagnostic gold standard [8]. In Brazil, the prevalence of CT is still unclear since its notification to Brazilian public health agencies is not mandatory. Previous studies in Brazilian pregnant and non-pregnant women have demonstrated that CT infections ranged between 4% and 13%, using a variety of diagnostic tests [9-12]. This variation is more evident when the prevalence of CT is investigated in women who were candidates for in vitro fertilization (IVF) at a public institution. In São Paulo, Brazil, the prevalence of CT with samples obtained from cervical swabs was 1.1% (two out 176; 95% CI: 0.03-4) [1], while in Manaus, Amazonas, it was 52.8% (56 out 106; 95% CI: 43.4-62) [13]. Of note, in a similar region of Amazonas, Marajó Island, the prevalence was reduced to 4% (16 out of 393; 95% CI: 2.5-6.5) [11].

The prevalence of CT in urine samples using the RT-PCR technique in private IVF clinics in Brazil is scant. Therefore, it seems appropriate to investigate the prevalence of CT infection in women attending the private sector IVF clinics. It is important to analyze the prevalence of CT infection in these patients in order to adjust treatments [1]. 

The objective of this study is to verify the prevalence of CT in infertile women attending a private infertility clinic in southern Brazil. As a secondary objective, we compared the prevalence of CT in women with primary or secondary infertility.

## Materials and methods

This study was submitted and approved by the Internal Review Board of Hospital de Clínicas de Porto Alegre, with the number 2020-0659, and registered with the Certificate of Ethical Appreciation CAAE: 42225620.2.0000.5327. All patients were contacted by the first author and gave their written consent allowing their electronic records to be reviewed for the study. 

Study design and setting

This cross-sectional study was conducted in a private infertility clinic in southern Brazil (Clínica De Reprodução Humana PROSER, Porto Alegre, Rio Grande do Sul, Brazil) between January 1, 2019, and August 30, 2021. Urinary RT-PCR for CT was the first part of the investigation of infertile couples. 

Participants

Consecutive women between 18 and 50 years old who attended the PROSER clinic for IVF and had the result of a morning urinary sample for RT-PCR for CT in their electronic record were included in the study. Those without RT-PCR for CT were excluded.

Variables

The main outcome was the detection or not of CT in the urine. Further variables were age, body mass index (BMI), time and type of infertility, and indication for in vitro fertilization (IVF) due to infertility factors; briefly, endometrial, male, ovarian, and tubal factors. Those with two or more concomitant factors were considered as "others". 

Data sources and measurements

Urinary RT-PCR for CT was performed using *Chlamydia trachomatis*/*Neisseria gonorrhea* CT/NG Abbott RealTime PCR, using M200 real-time system (Abbott Molecular Diagnostics, Des Plaines, USA). Women brought a first-morning urine sample in a sterile container, according to the manufacturer's instructions. The test has a sensitivity of 320 plasmid copies of CT target DNA per assay and specificity of no cross-reactivity with 111 organisms. The average time between sample collection and processing was six hours.

Bias

Data were collected using an electronic form and stored in an electronic spreadsheet (all from Google LLC, Mountain View, USA) using dropdown cells to reduce data collection bias.

Study size

The sample size was calculated using a 95% confidence interval for a proportion considering an expected proportion of 10% and a total width of the confidence interval of 10% (i.e., 95% CI: 5% to 15%), based on the literature [9]. With these inputs, at least 156 cases would be necessary.

Quantitative variables 

Women were divided into two groups: those with primary and secondary infertility, according to the World Health Organization (WHO) [14]. Age, BMI, and time of infertility were handled as continuous variables, and IVF indication was considered as categorical data. Categorical data for infertility was according to the WHO fact sheet on infertility [14].

Statistical methods

Statistical analysis was performed using parametric and non-parametric statistics according to normal distribution. Normal distribution was analyzed using the Kolmogorov-Smirnov test. If data did not have a normal distribution, the Mann-Whitney U test was used to compare age, time of infertility, and BMI between women with primary and secondary infertility. IVF indications were analyzed using chi-square for trend. The proportion of the sample (detected and non-detected CT in urinary samples) was analyzed using a 95% confidence interval. Data were analyzed using GraphPad InStat 3.06 for Windows (GraphPad Software, San Diego, USA).

## Results

A total of 203 consecutive women accepted to participate in the study. No one refused to participate.

The prevalence of CT in the study was 10.84% (22 out of 203 patients; 95%CI: 7.27-15.87). Patients with secondary infertility were older than those with primary infertility, and they had more ovarian and tubal factors compared to primary cases (Table 1).

**Table 1:** Characteristics of the studied population BMI - body mass index; CT: Chlamydia trachomatis ^a^ Mann-Whitney test; ^b^ Chi-test for trend; ​​​​​​​^c^ Fisher's exact test

Characteristics	Type of infertility		p-value
	Primary n=150	Secondary n=53	
Age in years: median (range)	37 (20-50)	39 (28-49)	0.004^a^
BMI	23.9 (18.3-35.2)	23.7 (18.8-36.7)	0.6^a^
Time of infertility in years: median (range)	3 (0.6-18)	3 (0.5-11)	0.2^a^
Infertility factors	n (%)	n (%)	p-value
endometrial	23 (15.3)	5 (9.4)	0.004^b^
male	37 (24.7)	4 (7.5)	
ovarian	31 (20.7)	13 (24.5)	
tubal	41 (27.3)	19 (35.8)	
others	18 (12)	12 (22.6)	
Detection of CT [95% CI]	16 (10.67) [6.72-16.73]	6 (11.32) [5.57-23.76]	1^c^

No difference was found comparing the incidence of CT in women with primary and secondary infertility (Table 1). 

## Discussion

The prevalence of CT infection in our study was 10.84% (95% CI: 7.27-15.87). This prevalence is in accordance with the rates reported in the USA (7.2% in women between 20-44 years old) [15], higher than those described in Campinas, São Paulo [1], but lower compared to Manaus, Amazônia [13]. Pantoja et al. found a prevalence of 1.1% (two out 176; 95% CI: 0.03-4) in infertile women candidates for IVF in public in vitro fertilization reference clinic in southeastern Brazil [1], while Freitas et al. found a higher prevalence of 52.8% (56 out of 106, 95% CI: 43.4-62) in the same population studied, i.e., infertile women attending an infertility clinic at a public university hospital [13]. Nevertheless, our results are similar to those published by others: at a primary health care clinic, 8.4% (65 out of 781; 95% CI: 6.5-10.4) [16]; in a multicentric nationwide study in Brazil on a sample of pregnant women 9.4% (311 out of 3,303; 95% CI: 8.4-10.5), and at an outpatient obstetrics and gynecology clinic at a university teaching hospital 11% (22 out 200; 95% CI: 7.3-16) [17]. Possible explanations for these discrepancies could be related to the method used, i.e., cervical swab instead of urine [12]; variations in the population may also contribute to these discrepancies. For instance, a northern Brazilian population from a public hospital [13], in contrast to a southern Brazilian population. Yeganeh et al. analyzed vaginal swabs from 400 pregnant women in Porto Alegre, Brazil [18], and they reported a CT prevalence of 9.2% (37 out of 400; 95% CI: 6.8-12.5), which is within the same confidence interval that we found and in the same city.

As a secondary objective, we compared the prevalence of CT in women with primary or secondary infertility. No difference was found comparing the incidence of CT in women with primary and secondary infertility (Table 1). As expected, patients with secondary infertility were older than those with primary infertility. Higher age is expected among patients seen in infertility clinics. From 1999 to 2000, the CT prevalence among the general female household population aged between 14 and 39 years was 4.3% (95% CI: 3.1-6.1%). As these women aged, the CT prevalence fell to 0.8% (22 out of 2,667; 95% CI: 0.3-1.8) from 2007 to 2008 among women between 26 and 39 years old [19]. It is possible that the 11% prevalence found in a study published by Garcês et al. (22 out of 200; 95% CI: 7.3-16) might be overestimated since the sample was derived from an outpatient obstetric and gynecologic clinic [17]. A large Dutch prospective trial (Netherlands Chlamydia Cohort Study - NECCST), to be concluded in 2022, will elucidate the late complications and prolonged time to pregnancy, as a proxy for reduced fertility, due to a previous CT infection [20].

The ovarian and tubal factors were the most prevalent etiology of infertility in our population. No difference was found when only ovarian and tubal factors were compared between women with primary and secondary infertility (Table 1). The ovarian and tubal factors are the most common causes of female infertility worldwide, and the prevalence of these factors has been similar over the years [21,22]. The prevalence of the male factor was 24.7% and 7.5% in patients with primary and secondary infertility, respectively, similar to other studies [22,23].

Low and high BMI have a detrimental effect on women's fertility outcomes [24,25]. In our sample, the mean BMI was within the normal range; however, extremes in the upper limit were identified. No difference was found between women with normal and abnormal BMI compared to the presence or not of CT (data not shown). These results are similar to those reported in other studies [26].

This study has some limitations. Sexual risk behaviors, such as a number of sexual partners, history of pelvic inflammatory disease, history of abortions, and previous treatment for sexually transmitted infections, were not evaluated. Our results are derived from a single private IVF clinic, reducing external validity. 

Positive aspects of the study are the proper sample size and the use of RT-PCR for CT diagnosis.

## Conclusions

In conclusion, no difference was found in the incidence of CT in women with primary and secondary infertility. This finding is important to alert health authorities about reviewing guidelines for screening all sexually active women to prevent infertility. The prevalence of 10.84% of CT in this private IVF clinic raises the importance of screening for sexually transmitted infections for proper treatment and achieving better IVF with embryo transfer outcomes. Further studies are necessary to evaluate the impact of treating these patients and their IVF outcomes.
